# The effects of nursing work environment on patient safety in Saudi Arabian hospitals

**DOI:** 10.3389/fmed.2022.872091

**Published:** 2022-07-22

**Authors:** Reem N. AL-Dossary

**Affiliations:** Department of Nursing Education, College of Nursing, Imam Abdulrahman Bin Faisal University, Dammam, Saudi Arabia

**Keywords:** patients’ safety, work environment, nurses, nurse managers, Saudi Arabia

## Abstract

**Purpose:**

This study aims to investigate the impact of the nursing work environment on patients’ safety in Saudi Arabian hospitals.

**Methods:**

This study used a cross-sectional design for collecting the data related to the nursing work environment and patients’ safety from nursing staff in Saudi Arabian hospitals. The survey questionnaire included in this study has two pre-validated questionnaires including practice environment scale-nursing work index questionnaire and hospital’s survey on patients’ safety developed by Surveys on Patients Safety Culture. The survey link was forwarded to HR administrators of 96 hospitals in Saudi Arabia, which included 72 public hospitals, 23 private hospitals, and one public-private hospital. Three hundred sixty-nine responses were received. After removing the incomplete responses, 357 responses were considered for the data analysis, in which *t*-tests and Pearson’s correlation techniques were adopted.

**Results:**

Strongest correlations were identified between resource adequacy and work area (*r* = 0.763, *p* < 0.01), “participation in management and leadership” and work area (*r* = 0.712, *p* < 0.01), “participation in management and leadership” and supervisor/managers’ approaches (*r* = 0.731, *p* < 0.01), and “nursing care and inter-disciplinary relationships and frequency of events” (*r* = 0.701, *p* < 0.01).

**Conclusion:**

The nursing work environment factors, especially participation, management and leadership, nursing care, inter-disciplinary relationships, and resource adequacy have to be improved in order to improve the patients’ safety.

## Introduction

The safety of patients in hospitals is one of the primary elements in healthcare systems, which can have a direct impact on patients’ health and well-being. Patients’ safety is often confused with patients’ conditions, but studies (1–3) have identified that patients’ safety is the process of ensuring the prevention of errors and negative effects on patients while providing healthcare services, i.e., mitigating or complete absence of adverse events resulting from healthcare interventions, but not from patients’ conditions. Adverse events during healthcare services are one of the top causes of deaths and disability in the world (4). It was identified that one in ten patients experience adverse events and get harmed while receiving healthcare services at hospitals in high-income countries (5), while such incidents in middle-low-income countries are very high. Every year, 134 million unsafe care events were recorded in hospitals in middle-low-income countries resulting in 2.6 million deaths per year (6). Considering the global statistics, four in ten patients are harmed during primary care, and it was identified that 80% of this harm could be prevented. Patients’ safety goals such as correct identification of patients, precise communication, administering the right medication, increasing the safety of care operations, decreasing risk of infections, etc. are few internationally accepted patient safety guidelines (7).

Most of these safety operations are administered by nurses who form the largest professionals group in the healthcare system, as they have the most direct contact with the patients in hospitals, and can significantly influence patients’ safety (8). Studies have identified significant interactions between nurses’ working environment and patients’ safety. A study conducted in South Korea (9) has identified significant correlations between clinical career, nursing work environment, and patient safety culture, and further analysis through regression modeling revealed 30.3% of missed nursing care leading to adverse events. A similar study (10) in both public and private hospitals revealed that staffing and resource adequacy, professional communication style, and nurses’ participation in hospital quality improvement activities were associated with higher levels of perceived patient safety. Furthermore, positive correlations between working environment elements such as leadership, nursing care, and resource adequacy; and patients safety elements such as working environment quality, communication, frequency of adverse events, and managers’ approaches (11, 12). However, a recent study focusing on the review of the effects of nursing work environment and patients’ safety has found the effects to be inconsistent in various studies, which may be attributed to different working environments in different hospitals and countries (13). In addition to the work environment, it was observed that nurses’ perception of safety levels and satisfaction with the work environment can also have a significant impact on patients’ safety at hospitals (9, 11, 14). Hospitals that promote high-quality care for patients safety, interdisciplinary professional relations, positive communication, professional care models, greater possibilities for professional development, and better working conditions, attract qualified and skilled nurses and are able to retain them, and this whole process was found to be having a positive impact on patients’ safety in the studies conducted in the United States and Europe (15, 16). Positive nurses’ work environment was also associated with a reduced mortality rate at hospitals (17), good patient experiences (18), and effective quality of care (19).

A recent integrative literature review (9) focusing on the relationship between the nursing work environment and patients’ safety has identified that the majority of the studies reported varying results, which may be attributed to the selection of different measurement methods and settings. Accordingly, different results can be observed from the previous studies. For instance, a poor nursing work environment was positively correlated with missed nursing care, which can have a serious impact on the patients’ safety (10). Similarly, in Middle East countries, workplace bullying was observed to indicate a poor nursing working environment, which had a major impact on the patients’ safety; however, providing training for counteracting bullying made nurses less worried, which led to an improved working environment and patients’ safety (20). Therefore, steps taken to improve the nursing work environment can lead to improved patients’ safety. Similarly, in another Middle East country, only 35.2% of the nurses surveyed reported positive levels of perceived patients’ safety, reflecting the positive aspects of the work environment such as staffing and resource adequacy, professional communication style, and nurses’ participation in hospital quality improvement activities being positively correlated with patients safety (11). Similarly, studies (21, 22) have reflected that although there is a small number of participants reporting a positive work environment, positive associations were identified among them in relation to patients’ safety while achieving higher job satisfaction levels.

Studies related to the nursing work environment in Saudi Arabia mostly focused on the work related-stress (23), relationships between work environment, job satisfaction, and nurses’ retention (24), challenges in the work environment (25), and leadership aspects in the work environment (26); while the focus on the impact of nursing work environment and patient’s safety is under-researched. Furthermore, with a rapid increase in the prevalence of chronic diseases (27, 28), and frequent epidemics such as severe acute respiratory syndrome (SARS), middle east respiratory syndrome (MERS), and the recent COVID-19 pandemic in Saudi Arabia, it is highly important that patients’ safety in Saudi Arabian hospitals have to be investigated in relation to various parameters, especially the nursing work environment. Considering the abovementioned factors, this study aims to investigate the impact of the nursing work environment on the patients’ safety in Saudi Arabian hospitals.

## Materials and methods

This study used a cross-sectional design for collecting the data related to the nursing work environment and patients’ safety from nursing staff in Saudi Arabian hospitals.

### Questionnaire design

The survey questionnaire included in this study has two pre-validated questionnaires including practice environment scale-nursing work index (PES-NWI) questionnaire (29) and hospital’s survey on patients’ safety (HSPS) developed by Surveys on Patients Safety Culture (30). The first part of the questionnaire was related to nurses’ demographics, which included ten questions. The second part of the questionnaire focused on the nursing work environment, which has ten items that need to be rated on a five-rating Likert scale. The first four items are related to the nurse’s participation in management and leadership; items five to eight are related to nursing care and inter-disciplinary relations, and; items nine and ten are related to adequate resources. The third part of the questionnaire is related to patients’ safety (HSPS), which has 32 items. The first 18 items are related to the nursing work area/unit; items 19 to item 20 are related to supervisor/managers’ approaches; items 23 to 28 are related to communication, and; items 29 to 31 are related to the frequency of events reported. Item 32 focuses on the overall safety grade. The reliability of the questionnaire was evaluated using the pilot study conducted with 23 nurses. The pilot study results achieved Cronbach’s alpha greater than 0.70 for all the items, reflecting good internal reliability and consistency (31). An online version of the survey was created using Google Surveys, and a link for the survey was generated.

### Sampling and participants

As the survey was targeted at nurses working in Saudi Arabian hospitals, the survey link was forwarded to HR a*dministrators* of 96 hospitals in Saudi Arabia, which included 72 public hospitals, 23 private hospitals, and 1 public-private hospital. Considering the total nursing workforce of 196,701 in Saudi Arabia (32), an estimated sample was calculated using Cochran’s formula (33), at 95% CI and 5% of margin of error, giving an estimated sample of 384 participants. A total of 369 responses were received. After removing the incomplete responses, 357 responses were considered for the data analysis.

### Data analysis

Data were analyzed using SPSS version 20.0, and various statistical techniques including *t*-tests, Pearson’s correlation, and hierarchical regression were used. Missing data were removed in order to avoid any bias in analyzing the results. The primary outcome of the analysis is the correlation between the nursing work environment and patients’ safety.

### Ethical considerations

Informed consent was taken from all the participants using the checkbox option before starting the survey, where the users are provided with information about the study. The anonymity of the participants is ensured in this study. Ethical approval for the study was received from the Ministry of Health, Saudi Arabia.

## Results

As shown in Table 1, the majority of the participants were females, representing 60.5% of the total participants. The majority of the participants (64.7%) were qualified with a bachelor’s degree in nursing followed by 30.5% diploma holders, 4.2% master’s degree holders in nursing; and only two participants were having PhD in nursing. The majority of the participants were below 50 years of age; represented by 34.9% aged between 20 and 29 years, 42.3% aged between 30 and 39 years, and 15.1% aged between 40 and 49 years. Only 7.7% of the participants were aged between 50 and 59 years, and one participant was aged more than 59 years. The majority of the participants had more than 6 years of work experience, representing 66.9% of the total participants. In addition, 27.2% of participants had 1–5 years of experience; while 5.9% of participants had work experience of less than a year. Among the total participants, a majority (71.4%) were expatriate (non-Saudi) workers; 73.9% were staff nurses, and 26.1% were nurse managers. Furthermore, 23.8% of participants were working in primary healthcare centers, followed by 19% in regional public hospitals, 19% in specialized public hospitals, 14.3% in university hospitals, and another 14.3% in military hospitals reflecting a good distribution of participants across different hospital settings. Moreover, 38.1% of participants were from Eastern Region, 19% from Western Region, 19% from Southern Region, and 23.8% from Northern Region, reflecting a good representation of nurses across Saudi Arabia.

**TABLE 1:** Participants’ demographic information.

Demographic characteristics	*N*	Relative frequency
**Gender**		
Male	141	39.5%
Female	216	60.5%
**Education**		
Diploma	109	30.5%
Bachelor’s degree	231	64.7%
Master’s degree	15	4.2%
Doctorate	2	0.5%
**Age (years)**		
20–29	21	34.9%
30–39	225	42.3%
40–49	69	15.1%
50–59	41	7.7%
>59	1	0%
**Work experience**		
<1 years	11	5.9%
1–5 years	97	27.2%
6–10 years	67	18.8%
11–15 years	126	35.2%
16–20 years	29	8.1%
>20 years	27	7.6%
**Nationality**		
Saudi	102	28.5%
Non-Saudi	255	71.4%
**Role**		
Nurse manager	93	26.1%
Nurse	264	73.9%

All the work environment subscales were identified to be less effective. The overall mean rating for the “participation in management and leadership” was identified to be 2.4 out of 5; similarly, a 2.4 mean rating was identified for nursing care and inter-disciplinary relationships; and resource adequacy was poorly rated with a mean rating of 2.04; indicating an average nursing work environment in Saudi Arabian hospitals. To analyze further, the differences between the nurse managers and staff nurses in relation to their perceptions of the working environment were analyzed using *t*-tests, as shown in Table 2. No major differences were observed between the nurse managers and staff nurses in relation to the “participation in management and leadership” and “nursing care and inter-disciplinary relationships” subscales. However, in relation to resource adequacy, nurse managers believed that there are adequate resources (mean = 2.45); while nurses believed that there is a shortage of resources (mean = 1.85), especially in relation to the number of staff nurses required to meet the demand of providing quality care to the patients. Thus, significant difference of opinions was identified between nurse managers and staff nurses in relation to adequate resources (*t*-value = 2.7568; *p* = 0.0061, *p* < 0.05).

**TABLE 2:** Nurse managers’ and nurses’ perceptions of the practice environment.

	Nurse managers		Nurses		*t*	*p*
		
	Mean	SD	Mean	SD		
Participation in management and leadership	2.46	1.63	2.30	1.19	1.0066	0.3148
Focus on nursing care and inter-disciplinary relationships	2.41	1.52	2.31	1.02	0.7086	0.4790
Adequate resources	2.45	1.08	1.89	1.85	2.7568	0.0061*

* Statistically significant difference.

All the subscales related to the patients’ safety were rated to be better or good. The participants’ perceptions about the work area were identified to be average (mean = 2.55), while the supervisor/managers’ approach (mean = 2.85), and communication (mean = 3.03) were rated to be good. However, the frequency of events being reported was identified to be higher (mean = 2.82), which is a cause of concern. To further analyze the results, *t*-tests were carried out to identify if there are any differences in the opinions of nurse managers and staff nurses as shown in Table 3. Significant differences were observed with respect to subscales work area (*t*-value = 2.4849, *p* = 0.0134, *p* < 0.05), communication (*t*-value = 2.0876, *p* = 0.0375, *p* < 0.05), and frequency of events (*t*-value = 3.2870, *p* = 0.0011, *p* < 0.05) being reported. Nurse managers perceived all aspects of patients’ safety including work area (mean = 2.94), supervisor/managers’ approach (mean = 2.97), and communication (mean = 3.27) to be good and very good; staff nurses perceived them to be slightly good. While nurse managers perceived different instances of frequency of adverse events to be low (mean = 2.68), nurses perceived them to be higher (mean = 3.23).

**TABLE 3:** Nurse managers’ and nurses’ perceptions of patients’ safety.

	Nurse managers		Nurses		*t*	*p*
		
	Mean	SD	Mean	SD		
Work area/unit	2.94	1.91	2.42	1.67	2.4849	0.0134*
Supervisor/Manager approach	2.97	1.08	2.81	1.84	0.7915	0.4292
Communication	3.27	1.12	2.95	1.32	2.0876	0.0375*
Frequency of events reported	2.68	1.49	3.23	1.35	3.2870	0.0011*

*Statistically significant difference.

The analysis of the relationship between the nursing work environment and patients’ safety subscales is presented in Table 4. The results revealed that significant positive correlations existed between all subscales of nursing work environment and patients’ safety. The strongest correlations were identified between resource adequacy and work area (*r* = 0.763, *p* < 0.01), “participation in management and leadership” and work area (*r* = 0.712, *p* < 0.01), “participation in management and leadership” and supervisor/managers’ approaches (*r* = 0.731, *p* < 0.01), and “nursing care and inter-disciplinary relationships” and frequency of events (*r* = 0.701, *p* < 0.01).

**TABLE 4:** Correlations between practice environment subscales, and patients’ safety subscales using Pearson’s product-moment.

	Work area/unit	Supervisor/Managers’ approach	Communication	Frequency of events reported
Participation in management and leadership	0.712**	0.731**	0.690**	0.651**
Focus on nursing care and inter-disciplinary relationships	0.671**	0.574**	0.686**	0.701**
Adequate resources	0.763**	0.541**	0.638**	0.682**

**Correlation is significant at the 0.01 level (two-tailed).

## Discussion and conclusion

The work environment subscales in Saudi Arabian hospitals reflected fairly better conditions as perceived by overall participants, which are inconsistent with studies (11–14), but working conditions identified in Europe and the United States (15, 16) were identified to be much better. Studies in Saudi Arabia identified a high prevalence of work-related stress in the nursing environment (9, 10, 23) as well as emotional exhaustion and poor job satisfaction, which can be related to the work environment (24), thus indicating the lack of effective nursing work environment in Saudi Arabian hospitals, which may significantly impact patients’ safety. Furthermore, the quality of resources along with various variables reflected poor working environments for nurses in Saudi Arabia as identified in (20, 25) and the Middle East (11, 21) inconsistent with findings in this study. Accordingly, all the nursing work environment subscales were identified to be fairly better by both nurse managers and staff nurses, as there were no significant differences identified. However, in relation to resource adequacy, nurse managers indicated fairly better conditions, while staff nurses reflected poor conditions in Saudi Arabian hospitals inconsistent with (25). Therefore, there is a need to improve the nursing work environment in Saudi Arabian hospitals on par with the conditions in the countries like Europe and the United States, which result in increased job satisfaction, better patient outcomes, and patients’ safety (15, 16).

Focusing on the patients’ safety subscales all the participants’ reflected fairly good conditions. While the work area reflected fairly better conditions, all other scales including supervisor/managers’ approach, communication, and frequency of events being reported reflected fairly good conditions similar to previous studies (8–14, 17, 18). However, significant differences were observed in relation to patients’ safety between nurse managers and staff nurses. Nurse managers perceived work areas and communication to be good, while nurses reflected slightly contrasting views. It may be due to the fact that the work area operations and communications are managed by nurse managers according to their styles of leadership or approaches, as a result, they tend to feel that they are correct. Further supporting the analysis no differences were identified in relation to managers’ approach to handling patients’ safety, while both nurse managers and staff nurses perceived it to be good, but not the best. Accordingly, nurse managers perceived the frequency of different adverse events to be low, while staff nurses perceived them to be high, indicating the poor reporting approaches adopted in Saudi Arabian hospitals in similar to findings in (25).

Significant correlations were identified between the nursing work environment and patients’ safety subscales. As discussed previously, adequate resources are positively correlated with work area, indicating that lack of sufficient resources such as nursing personnel and quality resources can affect communication and result in the occurrence of adverse events. As Saudi Arabian hospitals include expatriates from different countries, communication could be an issue, which may lead to the occurrence of adverse events. Nursing care and inter-disciplinary relationships were identified to be strongly correlated with work area, communication, and occurrence of adverse events, which indicates that a collaborative and supportive working environment under good leadership is essential for improving the patients’ safety. Accordingly, participation in management and leadership was strongly correlated with all scales of patients’ safety. Therefore, the findings have revealed that the nursing work environment is positively correlated with patients’ safety; and the findings of poor nurses working conditions in Saudi Arabian hospitals will impact patients’ safety. All the areas in the nursing work environment, especially participation, management and leadership, nursing care, inter-disciplinary relationships, and resource adequacy have to be improved in order to improve the patients’ safety.

These findings can have various implications. First, the findings in this study contribute to the lack of literature in Saudi Arabian healthcare focusing on the issue of patients’ safety and the factors influencing it. Second, the findings such as poor nursing work environment and the results related to specific subscales can help decision-makers in improving the working conditions in Saudi Arabian hospitals, reflecting the practical implications. This study also has a few limitations. First, though there are different factors influencing patients’ safety, the only nursing work environment is considered in this study. Second, the sample collected in this study also represented different regions, which is considered to be low, given the number of different hospitals, their working environments, and the number of nurses/resources. These limitations can guide future research in linking the nursing work environment in different hospitals, and their effect on patients’ safety.

## Data availability statement

The raw data supporting the conclusions of this article will be made available by the authors, without undue reservation.

## Ethics statement

The studies involving human participants were reviewed and approved by Ministry of Health, Saudi Arabia. The patients/participants provided their written informed consent to participate in this study.

## Author contributions

The author confirms being the sole contributor of this work and has approved it for publication.

## Conflict of interest

The author declares that the research was conducted in the absence of any commercial or financial relationships that could be construed as a potential conflict of interest.

## Publisher’s note

All claims expressed in this article are solely those of the authors and do not necessarily represent those of their affiliated organizations, or those of the publisher, the editors and the reviewers. Any product that may be evaluated in this article, or claim that may be made by its manufacturer, is not guaranteed or endorsed by the publisher.
